# Endometriosis: Challenges in Clinical Molecular Diagnostics and Treatment

**DOI:** 10.3390/ijms26093979

**Published:** 2025-04-23

**Authors:** Pedro Rosendo-Chalma, Erick Nicolás Díaz-Landy, Verónica Antonio-Véjar, Jonnathan Gerardo Ortiz Tejedor, Claudia Reytor-González, Daniel Simancas-Racines, Gabriele Davide Bigoni-Ordóñez

**Affiliations:** 1 Laboratorio de Virus y Cáncer, Unidad de Investigación Biomédica en Cáncer of Instituto de Investigaciones Biomédicas-Universidad Nacional Autónoma de México (IIB-UNAM), Mexico City 14080, Mexico; prosendo.chalma@gmail.com; 2 Unidad Académica de Salud y Bienestar, Carrera de Bioquímica y Farmacia, Universidad Católica de Cuenca, Cuenca 010101, Ecuador; jonnathan.ortiz@ucacue.edu.ec; 3 Unidad Académica de Posgrado, Maestría en Diagnóstico de Laboratorio Clínico y Molecular, Universidad Católica de Cuenca, Cuenca 010101, Ecuador; 4 Unidad de Ginecología y Obstetricia, Hospital Santa Inés, Cuenca 010107, Ecuador; nicodiazlandy@gmail.com; 5 Ginecología y Obstetricia, Universidad del Azuay, Cuenca 010204, Ecuador; 6 Laboratorio de Virología y Patología Traslacional, Facultad de Ciencias Químico Biológicas, Universidad Autónoma de Guerrero, Chilpancingo 39090, Mexico; 11335@uagro.mx; 7 Centro de Investigación en Salud Pública y Epidemiología Clínica (CISPEC), Facultad de Ciencias de la Salud Eugenio Espejo, Universidad UTE, Quito 170527, Ecuador; claudia.reytor@ute.edu.ec; 8 Carrera de Laboratorio Clínico, Facultad de Ciencias Médicas, Universidad de Cuenca, Cuenca 010201, Ecuador

**Keywords:** endometriosis, molecular markers, diagnostic, treatment

## Abstract

Endometriosis is a chronic disease affecting approximately 10% (190 million) of women and girls of reproductive age worldwide. It is associated with a variety of often debilitating symptoms, including severe pelvic pain, pain during intercourse, bowel movements and/or urination, bloating, nausea, fatigue, risk of infertility, as well as depression and anxiety in some cases. This review summarized the pathogenesis of endometriosis and the criteria for clinical diagnosis, proposed a panel of potential biomarkers for predictive molecular diagnosis, as well as choice of treatments for pain and infertility management.

## 1. Introduction

Endometriosis is a chronic benign gynecological disease characterized by the presence of endometrial-like tissue (glands and stroma) outside the uterine cavity [1,2]. This ectopic tissue commonly implants itself in pelvic organs such as the ovaries and fallopian tubes, but it can also affect extra-pelvic organs including the liver, lungs, intestines, skin, and subcutaneous tissues [3]. The most frequent symptoms experienced by patients with endometriosis include severe pelvic pain, dysmenorrhea, dyspareunia, and infertility, significantly impacting their professional, social, and sexual lives [4]. Endometriosis is frequently underestimated and undervalued in the medical community, causing women affected by this condition to continuously seek care [5,6]. Additionally, delays in diagnosing and treating endometriosis are partly due to medical professionals normalizing menstrual pain, as well as the misconception that surgery is mandatory for a definitive diagnosis [7].

### Epidemiology of Endometriosis

Endometriosis affects approximately 10% of women of reproductive age worldwide, equivalent to more than 176 million cases [8]. However, the lack of a standardized and non-invasive diagnostic method, together with the stigma associated with the symptoms, contributes to significant delays in diagnosis. These delays range from 4 to 11 years [9], in some cases extending up to 13 years, further exacerbating the situation [10]. These difficulties underline the urgency of improving diagnostic strategies and global awareness, especially considering the wide variability in prevalence reported in different regions.

At a global level, the prevalence of endometriosis varies considerably depending on the methods of diagnosis and the populations studied. For example, a global analysis based on multiple approaches reported rates as low as 0.05%, reflecting the inherent limitations of heterogeneous diagnostic criteria and characteristics of the populations analyzed [11]. However, it is estimated that the actual prevalence is likely much higher, particularly in women with specific symptoms such as chronic pelvic pain or infertility.

At a regional level, Europe demonstrates a range of prevalences influenced by the country and diagnostic method used. In Italy, the prevalence was 3.2% in women over 30 years old diagnosed by surgery or ultrasound [12], while in Germany, lower rates were reported in women over 14 years old, ranging from 0.5% and 0.7%, when diagnosed via laparoscopy or clinical symptoms, respectively [13]. North America reports a prevalence of 4.5% in women aged 18–45 years, based on self-reporting and confirmation via laparoscopy [14]; this prevalence increased to 8.0% when considering women aged 15–49 years diagnosed via laparoscopy or hysterectomy [15].

In Asia, Jordan reported a high prevalence of 13.7% in women aged 16–50 years using laparoscopy [16]. In Oceania, studies in Australia showed a prevalence of 7.8% in women born between 1945 and 1975 [17]. This increased to 11.4% when considering young women aged 18–23 diagnosed via a combination of laparoscopy, medical records, and self-reporting methods [18]. In Latin America, notably high prevalence rates were reported in Brazil, with 16.3% of women aged 21–44 undergoing laparoscopic sterilization [19]. In Africa, a study in Nigeria reported a prevalence of 10.9% in women aged 21–60 based on pathology reports [20]. Finally, in the Middle East, Saudi Arabia reported a prevalence of 11.1% in women diagnosed via laparoscopy [21]. When considered together, these figures illustrate how factors such as access to advanced diagnostic methods including laparoscopy, awareness of the disease, and medical practices significantly influence prevalence estimates. Standardization of diagnostic criteria and improved access to healthcare are critical to more accurately understand the global burden of this disease.

## 2. Clinical Aspects of Endometriosis

### 2.1. Pathogenesis

Several theories have been proposed regarding the origin of endometriosis; however, only three of them explain how the endometrial epithelium implants and develops in other organs. The first theory, described by Sampson, is based on retrograde menstruation with reflux to the peritoneum through the fallopian tubes [22]. This theory is supported by studies, with evidence of this reflux in almost 90% of patients; however, only 10–15% of these patients develop endometriosis [23]. The second theory is based on coelomic metaplasia, where totipotent cells in the peritoneum have the capacity to develop into endometrial epithelium, causing inflammation and fibrosis [24]. The third theory is described as embryonic rest, where the cells of Müllerian ducts are present in the peritoneum and throughout the body, which could explain pulmonary and diaphragmatic endometriosis [24]. At present, the most studied theory is that of immunology, where the immune system fails to recognize and eliminate misplaced endometrial cells outside the pelvic cavity [25].

Due to the limited understanding of the etiology of endometriosis, autologous or syngeneic female animal models have been employed. In these models, endometrial fragments derived from excised uterine horns are injected into the peritoneal cavity, resulting in the development of endometriotic lesions, increased vascular growth (angiogenesis), and reduced fertility [26,27,28]. These findings have supported the association between endometriosis and impaired fertility and have contributed to the identification of the HOXA10 gene as the first genetic factor associated with the disease [29,30]. It has been demonstrated that homeobox 10A (HOXA10) expression is reduced in women with endometriosis compared to healthy controls [31]. However, further research is needed to fully elucidate the underlying mechanisms involved in the pathogenesis of endometriosis.

### 2.2. Classification

The current challenge for all scientific societies studying endometriosis is classification. There are various classifications that encompass many findings from clinical, surgical, imaging, and fertility diagnostics; however, to date there is no consensus.

#### 2.2.1. Revised Classification by the American Society for Reproductive Medicine (r-ASRM)

The most widely accepted classification, due to its clinical simplicity, is that by the American Society for Reproductive Medicine (r-ASM), which was first described in 1985 and updated in 1996. However, the classification is debatable as it does not address the complexity and surgical features for large infiltrating lesions in the rectum, ileum, ureter, and vagina [32]. The classification is summarized as follows:

- Stage I: minimal, isolated implants without adhesions;
- Stage II: mild, superficial implants of less than 5 cm or on the surface of the peritoneum and ovaries;
- Stage III: moderate, multiple implants, that are superficial or invasive, with evident tubal or peri-ovarian adhesions;
- Stage IV: severe, multiple implants both superficial and deep, ovarian endometrium with extensive firm and membranous adhesions [32].

#### 2.2.2. ENZIAN Classification

Medical advancements in laparoscopic and minimally invasive surgeries have created an urgent need for a classification system that includes both surgical and clinical aspects. This will ensure adequate pre-surgical assessment, reduce surgical risk, and improve prognoses. Therefore, the #ENZIAN classification system was developed [33].

The potential advantage of this classification system is that it combines both invasive and non-invasive procedures which demonstrate strong correlation in the #ENZIAN classification system pre- and peri-operatively. This enables adequate surgical planning to predict, through imaging, the area to intentionally search for adhesions or endometriotic foci affecting the patient [34].

### 2.3. Clinical Symptoms and Diagnostic Examination

Current clinical approaches focus mainly on gynecological history. The clinical guideline for endometriosis of the European Society of Human Reproduction and Embryology (ESHRE) recommends that endometriosis is considered present based on a combination of the following symptoms: dysmenorrhea, dyspareunia, dysuria, dyschezia, rectal bleeding or haematuria, catamenial pneumothorax, cyclical cough, hemoptysis, fatigue, and infertility [35]. However, additional symptoms that are not currently included in the ESHRE guidelines could also be considered. These include heavy or irregular menstrual bleeding, spotting or intermenstrual bleeding, diarrhea or constipation associated with menstruation, fatigue, nausea, and pain located in the lower abdomen, back, or chest [36,37]. In addition, it is recommended that transvaginal ultrasound and Magnetic Resonance Imaging (MRI) are utilized to confirm the diagnosis of endometriosis. Nevertheless, endometriosis should not be ruled out if these diagnostic methods return a negative result [38]. Instead, for those patients that return a negative imaging result and do not respond to empirical treatment, the guidelines recommend offering laparoscopy for diagnosis and treatment of suspicious lesions [35].

### 2.4. Endometriosis Fertility Index (EFI)

The Endometriosis Fertility Index (EFI) was published in 2010 with the aim of estimating the likelihood of pregnancy in patients with endometriosis. The index combines predictive scores based on age, infertility time, previous pregnancies, tubal–ovarian function, and endometriosis classification following r-ASM. This classification is widely accepted by reproductive surgeons [39].

## 3. Molecular Aspects of Endometriosis

### 3.1. Genetic Profile

The precise genomic basis underlying the development of endometriosis remains unclear, as candidate genes identified by several studies have yielded conflicting results that have not been consistently replicated across independent populations [40]. Genome-wide association meta-analyses have identified variants in several genes, including *SRP14*, *HOXB9*, *IFNG*, *GSTM1*, *WNT4*, *ICAM1*, *CYP17A1*, *PPARG*, *IL6*, *TGFB1*, *FSHR*, *TP53*, *TRA2A*, *VEZT/FGD9*, and *GREB1*, demonstrating significant differences in expression potentially linked to endometriosis. However, many of these genes are also expressed in other cell types relevant to the disease’s pathogenesis, such as neuronal, immune, and epithelial cells [40,41,42,43,44,45]. Therefore, further research is necessary to identify additional genetic variants that contribute to the pathogenesis of endometriosis.

### 3.2. Epigenetic Profile

Epigenetic modifications may play an important role in the pathogenesis of endometriosis, with potential implications for disease diagnosis, prognosis, and treatment. For instance, hypermethylation of promoter regions has been reported in genes such as progesterone receptor B [46], *E-cadherin* [47], and *HOXA10* [48]. Conversely, hypomethylation within the promoter of the cyclooxygenase-2 (*COX-2*) gene has been associated with elevated expression levels in both eutopic and ectopic endometriotic tissues [49,50]. Additionally, hypomethylation of the estrogen receptor beta (*ERβ/ESR2*) promoter region has been reported in activated endometriotic tissue [51,52], and increased *ERβ* expression has been linked to disease severity [53]. Interestingly, although promoter methylation is generally known to correlate with gene silencing or downregulation [54], the role of DNA methylation in endometriosis remains somewhat controversial. For example, hypermethylation of the steroidogenic factor-1 (*SF-1*) promoter region has been observed along with significantly elevated SF-1 mRNA levels in endometriotic stromal cells [55,56]. Subsequent studies, however, have clarified that hypermethylation specifically within a CpG island extending from exon 2 to intron 3 of the *SF-1* gene—distinct from the promoter—actually activates gene expression. This raises the hypothesis that this region may contain a silencer whose suppression, due to hypermethylation, results in increased *SF-1* expression [55,57].

In addition to DNA methylation, non-coding RNAs (ncRNAs)—functional RNA molecules transcribed from DNA but not translated into proteins—may also influence endometriosis development [58]. Several ncRNAs identified as dysregulated in endometriosis target mRNAs involved in diverse biological processes. For example, miR-20a and miR-148a modulate hypoxic injury; miR-21, miR-93, miR-199a-5p, miR-210, and miR-520g affect tissue remodeling and angiogenesis; miR-10b, miR-29c, miR-100, miR-145, miR-183, miR-202, miR-210, and miR-451 regulate cell survival and proliferation; miR-302a and miR-542-3p are involved in inflammation; miR-142-3p, miR-23, and miR-135 modulate steroidogenesis [58,59,60,61]. Given the multifactorial nature of endometriosis, identifying a panel of ncRNA biomarkers could improve predictive capacity and diagnostic accuracy for this disease.

### 3.3. Immunological and Inflammation Profile

An aberrant immune response in a peritoneal environment appears to play a crucial role in the proliferation of ectopic endometrial cells. For instance, secretion of chemokines and cytokines such as CCL-2 (MCP-1), CSF-1, IL-8, IL-1α, IL-1β, and RANTES contributes to the recruitment and polarization of macrophages from an M1 to an M2 phenotype within the endometriotic microenvironment. These M2-polarized macrophages subsequently secrete factors such as semaphorin 3A (Sema3A), nerve growth factor (NGF), and vascular endothelial growth factor (VEGF), promoting angiogenesis and participating in neuroangiogenesis associated with endometriotic lesions [62,63,64]. Furthermore, inflammatory cytokines, including IL-6, IL-8, CA-125, and TNF-α, are consistently elevated in women with endometriosis, suggesting their involvement in disease progression and highlighting their potential utility as biomarkers for non-invasive diagnosis [65,66].

### 3.4. Hormonal Profile

Endometriosis is an estrogen-dependent disease in which follicle-stimulating hormone (FSH) and aromatase are involved in estrogen production. Estrogen plays a fundamental role in the growth and proliferation of endometriotic tissue [67,68,69].

### 3.5. Angiogenic Profile

Angiogenesis, the process of new blood vessel formation, is essential for the growth and survival of endometriotic lesions [70,71,72]. In endometriosis, hypoxic conditions prevent the proteasomal degradation of hypoxia-inducible factor-1α (HIF-1α), thereby promoting the expression of VEGF, a critical factor in hypoxia-induced angiogenesis [73,74].

### 3.6. Invasive and Migratory Profile 

Hypoxia and estrogen have been reported to activate the epithelial–mesenchymal transition (EMT) through distinct pathways involving TGF-β and Wnt signaling, promoting cell proliferation and migration [75,76]. Additionally, proinflammatory factors such as TNF-α, IL-1β, and lipopolysaccharide (LPS), in combination with hypoxia-inducible factor-1α (HIF-1α), have been shown to enhance EMT, increase the motility of endometriotic epithelial cells [77], and induce angiogenic activity in endometriotic tissues [78,79]. These mechanisms could explain the survival and persistence of endometriotic lesions in the peritoneal cavity.

### 3.7. Fibrosis Profile

Fibrosis in endometriosis is a complex, multi-step process driven by chronic inflammation, EMT, fibroblast-to-myofibroblast transdifferentiation (FMT), and extracellular matrix (ECM) remodeling. The key molecular factors involved include transforming growth factor-beta (TGF-β), cyclooxygenase-2 (COX-2), connective tissue growth factor (CTGF), Notch1 signaling, sphingosine-1-phosphate (S1P), inflammatory cytokines such as interleukin-6 (IL-6), oxidative stress, and dysregulated matrix metalloproteinase (MMP) activity [80,81]. A deeper understanding of these mechanisms could enable the development of targeted antifibrotic therapies aimed at reducing disease progression and adhesion formation.

**Figure 1:**
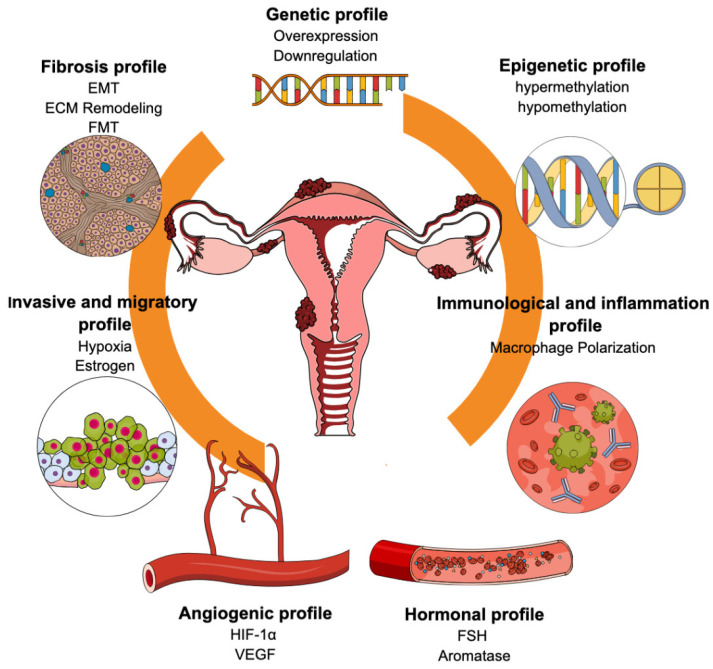
Schematic representation of the complex interplay between relevant profiles contributing to the understanding of endometriosis pathogenesis.

Figure 1 schematically summarizes the most representative molecular profiles of endometriosis that help understand its pathogenesis.

Because the etiology and pathogenesis of endometriosis are still far from being fully elucidated, animal and cellular models have provided valuable insights into the molecular processes involved. Additionally, studies employing integrated bioinformatics analysis have identified clusters of differentially expressed genes (DEGs) associated with cell migration, adherens junctions, and the HIF-1 signaling pathway [82,83,84]. However, because the molecular mechanisms underlying endometriosis pathogenesis closely resemble those activated during carcinogenesis, their potential as specific diagnostic biomarkers is significantly limited.

In this review, we summarized and critically analyzed biomolecules (DNA, RNA, and proteins) that have been reported in the literature as altered in patients with endometriosis compared to healthy controls. These biomolecules, identified across various studies, hold potential as diagnostic, prognostic, or treatment–response biomarkers for endometriosis. For example, Table 1 lists biomarkers including mutations, polymorphisms, epigenetic modifications in DNA, as well as changes in RNA and protein expression levels, all of which have diagnostic potential for endometriosis. Similarly, Table 2 summarizes biomarkers reported to have prognostic relevance, contributing to the understanding of disease progression. Finally, Table 3 presents a panel of miRNA biomarkers indicative of a favorable response to endometriosis treatment.

## 4. Endometriosis Treatment

The mechanism by which endometriosis causes pain remains unclear; the theory of hormonal stimulation by implants, local bleeding, and nerve infiltration may explain this phenomenon [35].

Therefore, treatment of endometriosis requires multidisciplinary management with several objectives, including pain and infertility.

### 4.1. Treatment Associated with Pain

- The first line of treatment is non-steroidal anti-inflammatory drugs (NSAIDs) and non-steroidal analgesics. According to the Cochrane Gynecology and Fertility Group’s Specialized Register of Controlled Trials, all NSAIDs are confirmed to be more effective than any other non-steroidal analgesics [110]. However, isolated use of NSAIDs does not prevent recurrence; therefore, management of acute symptoms is recommended [111].
- Hormonal treatment: The use of progestogen-only or combined oral contraceptives, as well as vaginal, transdermal, or injectable contraceptives significantly reduce dysmenorrhea and dyspreunia and substantially improve patients’ quality of life [112]. Likewise, the use of a progestogen-releasing intrauterine system and subdermal implants are a viable option for patients with pain associated with endometriosis [113].
- Gonadotropin-releasing hormone (GnRH) antagonists and agonists are used as second- and third-line treatments due to their side effects [114]. However, combined therapy, using an oral contraceptive and GnRH agonist currently approved in Europe, is an appropriate alternative for pain control and possible disease progression without causing the side effects of pure GnRH agonists or antagonists [114].
- Surgery is considered if other medical treatments fail to control pain. When surgery is performed, all endometriotic foci should be excised or ablated in a single surgical procedure [115]. Current guidelines (ESHRE) recommend that each patient is assessed individually to choose the treatment option that will most improve their quality of life, whether it be clinical or surgical treatment [115].

### 4.2. Treatment for Endometriosis Related Infertility

Current guidelines recommend that surgery for endometriosis-related fertility issues should be directed by associated symptoms, such as pain, dyspareunia, and infertility, to improve both patient quality of life and fertility rates [115]. At this point, medications such as GnRH antagonists or agonists are contraindicated as treatment for endometriosis and fertility [116].

Surgery may improve pregnancy rates in patients with endometrioma or damage in the fallopian tube. However, most patients will be candidates for ovarian stimulation or highly complex procedures to achieve pregnancy. Therefore, surgery should only be indicated by assessing the risks and benefits for each patient [117].

## 5. Conclusions

At present, molecular methods have provided important insights into some of the mechanisms underlying the origin of endometriosis. However, these mechanisms do not follow a consistent pattern across all patients, meaning imaging, physical examination, and surgical techniques remain essential for diagnosis. In this review, we focused on identifying biomarkers derived from comparative analyses between patients with and without endometriosis that can also be detected through non-invasive methods. Specifically, we highlighted biomarkers with potential applications for early clinical detection of the disease (Table 1), biomarkers to assess the risk of disease progression and infertility (Table 2), and biomarkers indicative of a favorable treatment response (Table 3). Nonetheless, additional research is needed to develop a reliable predictive model capable of calculating the probability that an individual either has or is susceptible to developing endometriosis based on measured biomarker levels.

## Figures and Tables

**Table 1:** Biomolecules as molecular markers for the diagnosis of endometriosis.

Biomolecule	Biomarker	Sample Type	Detection Method	Reference
DNA	Somatic mutations in *PIK3CA*, *KRAS*, *FBXW7*, *PPP2R1A*, *PIK3R1*, and *ARID1A* were frequent in endometriosis, and the mutation status of *ARID1A* leads to loss of its function and is a key event in the malignant transformation of endometriosis.	Tissue (laser microdissection)	Whole exome and targeted gene sequencing	[85,86,87]
DNA	Hypermethylation of several CpG sites in the genes *SLC10A6*, *MACROD1*, *EMX2*, and *PRDM16*, as well as hypomethylation in the genes *bZNF423*, *PPFIA1*, *ATP101A1*, and *PI3KCG*, were related to the development and pathogenesis of endometriosis.	Endometrial tissue: ectopic and eutopic	Infinium Human Methylation 450 K BeadChip assays	[88]
RNA	Detection of JARID2 activity (through *EZH2* expression and *H3K27* trimethylation) and high miR-155 expression are diagnostic biomarkers of endometriosis.	Peritoneal fluid, blood, plasma, serum, or endometrial tissue	MALDI-TOF, ELISA, Luminex, FACs, Western blot, dot blot, immunoprecipitation, immunohistochemistry	[89]
RNA	Patients with endometriosis have been reported to have the following microRNA expression levels: ↑miR-125b-5p, ↑miR-150-5p, ↑miR-342-3p, ↑miR-451a, ↓miR-3613-5p, and ↓let-7b.	Serum	qRT-PCR	[90]
RNA	Endometriosis was diagnosed via the expression of the following microRNAs: ↑miRNA-125b-5p, ↑miR-150-5p, ↑miR-342-3p, ↑miR-145-5p, ↑miR-143-3p, ↑miR-500a-3p, or ↑miR-18a-5p.	Samples of blood, serum, or plasma.	qRT-PCR or microarray	[91]
RNA	High expression of lncRNA H19 was associated with clinical features of endometriosis, infertility, and bilateral ovarian lesions.	Tissue	qRT-PCR	[92]
RNA	hsa-miR-21 and hsa-miR-200b are highly expressed in patients with endometriosis.	Blood sample	qRT-PCR	[93]
Protein	↑CA19-9, ↑CA125, and ↑D-D.	Serum and plasma	Electrochemiluminescence immunoassay and Latex immunoturbidimetry	[94,95,96]
Protein	↑TGFBI	Plasma	ELISA	[97]
Protein	↑CA125, ↑CA72-4, ↓ HE4, ↑CA19-9, ↑E2, and ↑EMS	Serum	ELISA and chemiluminescence	[98,99,100]
Protein	↑MMP-2	Endometrial tissue	Immunohistochemistry (IHC)	[101]
Protein	↑MMP-2 and ↑MMP-9	Serum	ELISA	[102]
Protein	↓ERRα	Serum	ELISA	[103]
Protein	↑ER-α, ↑PR, ↑AR, and ↑Aromatase	Endometrial tissue	Immunohistochemistry (IHC)	[104]

↑ = high level of expression; ↓ = low level of expression.

**Table 2:** Biomolecules as molecular markers for the prognosis of endometriosis.

Biomolecule	Biomarker	Sample Type	Detection Method	Reference
DNA	Polymorphic variants in the *CIITA* gene may contribute to the development of endometriosis.	Pathological biopsy	Complex exome sequencing	[105]
DNA	*PTPN22* (C1858T) polymorphism may be a marker of predisposition for endometriosis.	Peripheral blood	Polymerase Chain Reaction–Restriction Fragment Length Polymorphism (PCR-RFLP)	[106]
DNA	rs2268613 polymorphism in the *PLGF* gene is associated with ↓PLGF plasma levels, and rs3025039 (C > T) polymorphism in VEGF and ↑VEGF plasma levels are associated with an increased risk of endometriosis.	Tissue	TaqMan SNP genotyping assay–Quantikine ELISA.	[107,108]
DNA	The 4q12 locus (rs17773813), located upstream of the *KDR* gene that encodes receptor 2 (VEGFR2) factor for vascular endothelial growth, is correlated with the risk of developing endometriosis.	Tissue	Whole-Genome Sequencing	[109]
RNA	Through logistic regression analysis, it was shown that ages below 40 years and overexpression of lncRNA H19 in ectopic endometrium are prognostic factors for endometriosis recurrence.	Endometrial tissues: ectopic and eutopic	qRT-PCR	[92]

**Table 3:** Biomolecules as molecular markers for the response to endometriosis treatment.

Biomolecule	Biomarker	Sample Type	Detection Method	Reference
RNA	A decrease in the level of miRNA-125b-5p and an increase in the levels of miR-150-5p and miR-3613-5p are indicative of a response to treatment for endometriosis, where treatment consists of: hormonal therapy, hormonal contraceptive, gonadotropin-releasing hormone agonist, and gonadotropin-releasing hormone antagonist.	Sample of blood, serum, or plasma.	qRT-PCR or microarray	[91]

## Data Availability

Not applicable.
